# Foot ulcers in diabetes mellitus patients – protein analysis of wound environment

**DOI:** 10.1186/1878-5085-5-S1-A74

**Published:** 2014-02-11

**Authors:** Lucie Vistejnova, Milena Kralickova

**Affiliations:** 1 Department of Histology and Embryology, Faculty of Medicine in Pilsen, Charles University in Prague, Pilsen, Czech Republic; 2 Biomedical Centre, Faculty of Medicine in Plzen, Charles University in Prague, Plzen, Czech Republic

**Keywords:** foot ulcers, chronic wound, inflammation, protein analysis

## 

Diabetes mellitus (DM) is associated with many health complications and foot ulcers are one of the escalating, which brings uncomfortable and painful lifestyle for DM patients. In severe cases, foot ulcers can lead to a lower extremity amputation or even death. The treatment of such affected patients results in a high economic load [1].

Immune system of DM patients shows improper function leading to chronic wound of foot ulcers. In a physiological wound healing, wound is cleared from bacteria by immune cells, keratinocytes perform re-epithelization, fibroblasts restore dermis matrix and endothelial cells ensure angiogenesis. All is perfectly driven by growth factors, cytokines and chemokines which are dozen in a sensitive balance. In the foot ulcers of DM patients, immune cells over-express pro-inflammatory cytokines and chemokines (IL-1, IL-6, TNF-α, CCL2, CCL3, CCL4, CXCL1, CXCL5, CXCL8) and are not able to clear wound bed from infection. Fibroblasts and keratinocytes have reduced proliferative, locomotive and secreting functions and are not able to renovate epidermis and dermis followed by lacking angiogenesis [2]. High levels of pro-inflammatory cytokines, chemokines and proteases produced by persisting bacteria and activated immune cells represent main factors supporting the improper healing of foot ulcers.

The standard treatment of diabetic ulcers includes optimization of glycemic control, extensive debridement, infection elimination, use of moisture dressings, and offloading high pressure. New approaches such as autologous skins transplantation, mesenchymal stem cell application or dressings containing growth factors started to be utilized [1]. The development of new treatments requires a multidisciplinary cooperation and a deep molecular-biological research. The identification of microbiological contamination is a common approach and serves for the development and the evaluation of efficient antibacterial preparations. The protein analysis of wound debris and wound exudates utilizing multiplex immunological methods represent a quite new method and can provide an overview of chronic wound protein content, which can bring information for the development of curing preparations focused on cytokine, chemokine or proteases function. The determination of protein composition of chronic wound further enables the monitoring of healing process and provides the data about efficacy of healing management.

Although the foot ulcers care is at high level and not all patients are judged to extremity amputation, the healing of foot ulcers in DM patients is a constant challenge. The field for development of an effective, economically and patient’s friendly medical preparation with doctor’s favorable application form is still open.
